# Patterns of antiemetic medication use during pregnancy: A multi-country retrospective cohort study

**DOI:** 10.1371/journal.pone.0277623

**Published:** 2022-12-01

**Authors:** Anat Fisher, J. Michael Paterson, Brandace Winquist, Fangyun Wu, Pauline Reynier, Samy Suissa, Matthew Dahl, Zhihai Ma, Xinya Lu, Jianguo Zhang, Colette B. Raymond, Kristian B. Filion, Robert W. Platt, Carolina Moriello, Colin R. Dormuth, for the Canadian Network for Observational Drug Effect Studies (CNODES) Investigators

**Affiliations:** 1 Faculty of Medicine, Department of Anesthesiology, Pharmacology and Therapeutics, University of British Columbia, Vancouver, British Columbia, Canada; 2 ICES, Toronto, Ontario, Canada; 3 Institute of Health Policy, Management and Evaluation, University of Toronto, Toronto, Ontario, Canada; 4 College of Medicine, University of Saskatchewan, Saskatoon, Saskatchewan, Canada; 5 Saskatchewan Health Quality Council, Saskatoon, Saskatchewan, Canada; 6 Center for Clinical Epidemiology, Lady Davis Institute, Jewish General Hospital, Montréal, Quebec, Canada; 7 Department of Epidemiology, Biostatistics and Occupational Health, McGill University, Montréal, Quebec, Canada; 8 Manitoba Centre for Health Policy, University of Manitoba, Winnipeg, Manitoba, Canada; 9 Department of Medicine, Cumming School of Medicine, University of Calgary, Calgary, Alberta, Canada; 10 Department of Medicine, McGill University, Montréal, Quebec, Canada; 11 Department of Pediatrics, McGill University, Montréal, Quebec, Canada; West China Second University Hospital of Sichuan University, CHINA

## Abstract

**Objective:**

To compare patterns in use of different antiemetics during pregnancy in Canada, the United Kingdom, and the United States, between 2002 and 2014.

**Methods:**

We constructed population-based cohorts of pregnant women using administrative healthcare data from five Canadian provinces (Alberta, British Columbia, Manitoba, Ontario, and Saskatchewan), the Clinical Practice Research Datalink from the United Kingdom, and the IBM MarketScan Research Databases from the United States. We included pregnancies ending in live births, stillbirth, spontaneous abortion, or induced abortion. We determined maternal use of antiemetics from pharmacy claims in Canada and the United States and from prescriptions in the United Kingdom.

**Results:**

The most common outcome of 3 848 734 included pregnancies (started 2002–2014) was live birth (66.7% of all pregnancies) followed by spontaneous abortion (20.2%). Use of antiemetics during pregnancy increased over time in all three countries. Canada had the highest prevalence of use of prescription antiemetics during pregnancy (17.7% of pregnancies overall, 13.2% of pregnancies in 2002, and 18.9% in 2014), followed by the United States (14.0% overall, 8.9% in 2007, and 18.1% in 2014), and the United Kingdom (5.0% overall, 4.2% in 2002, and 6.5% in 2014). Besides use of antiemetic drugs being considerably lower in the United Kingdom, the increase in its use over time was more modest. The most commonly used antiemetic was combination doxylamine/pyridoxine in Canada (95.2% of pregnancies treated with antiemetics), ondansetron in the United States (72.2%), and prochlorperazine in the United Kingdom (63.5%).

**Conclusions:**

In this large cohort study, we observed an overall increase in antiemetic use during pregnancy, and patterns of use varied across jurisdictions. Continued monitoring of antiemetic use and further research are warranted to better understand the reasons for differences in use of these medications and to assess their benefit-risk profile in this population.

## Introduction

Nausea and vomiting of pregnancy (NVP) is a common condition with evidence from North America and Europe that suggests it affects between 60% and 80% of pregnancies [1–3]. Symptoms usually start between 3 and 8 weeks of pregnancy and peak between 7 and 12 weeks [4]. In more severe cases, pharmacological treatment may be needed to prevent weight loss, dehydration, and electrolyte imbalance. NVP occurs at the time of organogenesis when the fetus is most susceptible to teratogens; therefore, exposure to certain medications used to manage NVP treatment may be associated with adverse pregnancy outcomes such as spontaneous abortion or congenital malformations [5–9].

Multiple pharmacotherapies are available to treat NVP. In Canada, the recommended first-line treatment and the only oral antiemetic medication approved for use during pregnancy is a sustained-release combination of doxylamine succinate, an antihistamine that blocks H_1_ receptors, and pyridoxine hydrochloride [10]. This combination product was approved for use by the regulatory agencies in the United States in 2013 [11] and the United Kingdom in 2018 [12]. It has been approved in Canada under the trade name Diclectin^®^ since 1983 [13]. Both doxylamine and pyridoxine were also available over-the-counter as immediate-release products before the sustained-release combination was approved. Other pharmacotherapies for NVP include H_1_-blocker antihistamines, such as phenothiazines, dopamine D2 antagonists, such as metoclopramide, and serotonin 5HT_3_ receptor antagonists, such as Ondansetron, which is prescribed off label for NVP [14,15]. Ondansetron [16] and other antiemetic medications [17,18] cross the placenta and have the potential to harm the fetus, especially during organogenesis. While early studies found no signals of harm associated with ondansetron [5,19,20], several studies published since 2015 have raised concerns about the safety of ondansetron during pregnancy [6,10]. Safety warnings have been issued also for other antiemetic drugs used in pregnancy. For example, in 2009, the United States Food and Drug Administration required the addition of a black box warning about the risk for tardive dyskinesia with chronic or high-dose use of metoclopramide [21]. Clinical practice guidelines have recommended doxylamine/pyridoxine as first-line pharmacotherapy for NVP for over 30 years in Canada and since at least 2004 in the United States [10,20,22–24]. Off-label prescribing of ondansetron has been reserved for women with severe NVP when other antiemetic combinations have failed [20,22]. Several epidemiological studies of trends in antiemetic utilization have shown that off-label use of ondansetron during pregnancy has increased considerably, particularly in the United States [25–31]. We are aware of only one multi-national study—a web-based survey—that described trends in the use of antiemetics during pregnancy [26].

## Methods

### Aim, design and data source

Our study aimed to describe the patterns of use of antiemetic medications during pregnancy in Canada, the United Kingdom, and the United States between 2002 and 2014. In a cohort study that followed and published elsewhere, we examined the association between ondansetron exposure during pregnancy and the risks of spontaneous abortion, stillbirth, and major congenital malformations [32].

We undertook a retrospective, population-based cohort study that used administrative healthcare data from five Canadian provinces (Alberta, British Columbia, Manitoba, Ontario, and Saskatchewan), the United Kingdom Clinical Practice Research Datalink (CPRD) [33], and the United States IBM^®^ MarketScan^®^ Commercial Research Database [34]. The study was conducted in a distributed fashion by seven research teams within the Canadian Network for Observational Drug Effect Studies (CNODES) [35] using a common research protocol.

Our research teams had access to Canadian population-based claims data that included physician billings, hospital discharge abstracts, and pharmacy dispensation claims. Emergency department data were only partially available. In Ontario, the pharmacy claims data were available only for women receiving social assistance. The CPRD contained the complete primary care electronic medical records (including medical diagnoses and prescriptions) for over 13 million individuals from over 680 general practices in the United Kingdom. CPRD data were linked to Hospital Episode Statistics data, which contain complete hospitalization records, and the CPRD Pregnancy Register [36]. The MarketScan Commercial Databases included healthcare information for families with private health insurance plans in the United States and contained inpatient and outpatient medical claims and outpatient pharmacy claims.

This study was made possible through data sharing agreements between CNODES member research centres and the respective provincial governments of Alberta, British Columbia, Manitoba (HIPC # 2015/2016–36), Ontario, and Saskatchewan. Research ethics board approvals were obtained at each participating institution, except at ICES in Ontario, where research ethics board approval was not legally required. The Research Ethics Board included: University of Calgary—Conjoint Health Research Ethics Board (Alberta), University of British Columbia—Clinical Research Ethics Board (British Columbia and MarketScan), University of Manitoba- Health Research Ethics Board (Manitoba), University of Saskatchewan—Biomedical Research Ethics Board (Saskatchewan), Biomedical Research Ethics Committee of the Centre Intégré universitaire de santé et de services sociaux (CIUSSS) du Centre-Ouest-de-l’île-de-Montréal (CPRD). ICES is a prescribed entity under Ontario’s Personal Health Information Protection Act (PHIPA). Section 45 of PHIPA authorizes ICES to collect personal health information, without consent, for the purpose of analysis or compiling statistical information with respect to the management of, evaluation or monitoring of, the allocation of resources to or planning for all or part of the health system. Projects that use data collected by ICES under section 45 of PHIPA, and use no other data, are exempt from REB review. The use of the data in this project is authorized under section 45 and approved by ICES’ Privacy and Legal Office.

The source population was 47 million women between the ages of 12 and 55 years who had at least 365 days of continuous enrollment in a health plan between April 1, 2001 and March 31, 2016. There were two exceptions: Alberta data were available from April 2008 onward, and MarketScan data were available from January 2006 until the end of study period. We constructed cohorts of pregnant women from this source population.

### Participants

We identified pregnancies using a modified version of a validated multi-step algorithm [37,38]. We extracted records of pregnancy outcomes—live births, stillbirths, spontaneous abortions, or induced abortions—between April 2002 and March 2016 from available data sources (S1 Table). We then constructed pregnancy episodes starting at the estimated date of last menstruation and ending on the date of birth or abortion. The date of last menstruation was estimated by subtracting gestational age, recorded or imputed, from the date of birth or abortion. If gestational age was not available, we used an algorithm developed by Hornbrook et al [38] to impute gestational age based upon median gestational age for each pregnancy outcome (70 days for spontaneous abortion or induced abortion, 196 days for stillbirth, and 280 days for live birth). Information on gestational age was missing and imputed for 48% of pregnancies in the Canadian data (all pregnancies in Alberta and 23%–37% in the other Canadian provinces), 100% of pregnancies in the MarketScan data, and 3% of pregnancies in the CPRD data. Next, we identified and eliminated overlapping records of pregnancies. We also excluded pregnancies with the last menstrual period (cohort entry) before 2002 (2008 for Alberta, 2007 for MarketScan) or after 2014. This was done to address the risk of selection bias resulting from the varying lengths of look-back periods for different pregnancy outcomes (i.e., shorter look-back period for abortions compared with the periods for live births). Dormuth and colleagues have published a detailed description of cohort construction [32].

### The use of antiemetic medications

We identified use of prescription antiemetic medications before and during pregnancies using pharmacy dispensing records in Canada and the United States and using prescription records of general practitioners in the United Kingdom. The included antiemetics were: ondansetron, combination doxylamine/pyridoxine, dimenhydrinate, chlorpromazine, metoclopramide, prochlorperazine, promethazine, granisetron, dolasetron, palonosetron, and meclizine. Combinations with ergot alkaloidse or promethazides were excluded and, given the absence of these data, we were unable to identify over-the-counter use of short-acting doxylamine or pyridoxine. We identified antiemetic use during the following exposure periods: (1) the 90 days before cohort entry, (2) the first trimester (up to 90 days after and including cohort entry), and (3) anytime during the pregnancy (from cohort entry to the pregnancy outcome date). Antiemetic use was defined as at least one day with antiemetic supply based on the dispensation or prescription date and the recorded days’ supply. When days’ supply data were unavailable (e.g., in Saskatchewan), they were estimated based on the quantity dispensed and the daily defined dose for each medication.

### Statistical analysis

We presented descriptive data on the prevalence of antiemetic use by pregnancy time interval, study jurisdiction, and year of conception. Data were presented as total number (percentages) of pregnancies. Women were eligible to enter the cohort multiple times.

## Results

We studied 3,856,041 pregnancies from seven jurisdictions (Table 1 and S2 Table). The most common pregnancy outcome was live birth (66.7%), followed by spontaneous abortion (20.2%), induced abortion (12.1%), and stillbirth (1.0%). Most pregnancies in this cohort were from Canada (45.5%) or the United States (44.9%). Pregnancy outcomes varied by country and by Canadian province but were especially heterogeneous for induced abortions.

**Table 1:** Pregnancies in included databases, by pregnancy outcome.

Pregnancy outcome	Country							Total (n = 3856041, 100.0%)
	Canada					United Kingdom	United States	
	Alberta (n = 420 296, 10.9%)	British Columbia (n = 763 442, 19.8%)	Manitoba (n = 256 378, 6.6%)	Ontario (n = 102 701, 2.7%)	Saskatchewan (n = 213 433, 5.5%)	CPRD (n = 369 744, 9.6%)	MarketScan (n = 1 730 047, 44.9%)	
Live births	269 628 (64.2)	488 073 (63.9)	179 290 (69.9)	53 696 (52.3)	158 479 (74.3)	266 521 (72.1)	1 155 136 (66.8)	2 570 823 (66.7)
Stillbirths	6422 (1.5)	4068 (0.5)	1179 (0.5)	563 (0.5)	1194 (0.6)	4679 (1.3)	18 747 (1.1)	36 852 (1.0)
Induced abortions	71 702 (17.1)	170 648 (22.4)	44 877 (17.5)	37 651 (36.7)	25 119 (11.8)	6748 (1.8)	113 827 (6.6)	470 572 (12.1)
Spontaneous abortions	72 544 (17.3)	100 653 (13.2)	31 032 (12.1)	10 791 (10.5)	28 641 (13.4)	91 796 (24.8)	442 337 (25.6)	777 794 (20.2)

Numbers are pregnancies (% from all pregnancies in the database), unless otherwise specified.

^a^CPRD, United Kingdom Clinical Practice Research Datalink.

Use of antiemetics varied by exposure period (Table 2) and was low (1.5%) prior to the start of pregnancy. During the first trimester, antiemetics were dispensed for 12.2% of pregnant women. Across jurisdictions, doxylamine/pyridoxine was the most commonly used antiemetic during the first trimester (6.9% of pregnancies), followed by ondansetron (3.7%). A total of 14.8% of pregnant women used antiemetic medications at any time during pregnancy; the most common were doxylamine/pyridoxine (7.9%) and ondansetron (4.8%).

**Table 2:** Use of antiemetic medication before and during pregnancy, by study site.

	Number of pregnancies	Number of pregnancies exposed to antiemetic medications during the study period (%), by exposure period		
		90 days prior to pregnancy	1st trimester of pregnancy	Anytime during pregnancy
**Any antiemetic medications**				
Alberta	420 296	6021 (1.4)	63 846 (15.2)	78 959 (18.8)
British Columbia	763 442	3806 (0.5)	108 813 (14.3)	118 573 (15.5)
Manitoba	256 378	3521 (1.4)	38 806 (15.1)	45 648 (17.8)
Ontario	102 701	1459 (1.4)	24 653 (24.0)	28 603 (27.9)
Saskatchewan	213 433	2460 (1.2)	33 360 (15.6)	38 757 (18.2)
*Canada overall*	1 756 250	17 267 (1.0)	269 478 (15.3)	310 540 (17.7)
United Kingdom (CPRD^a^)	369 744	2894 (0.8)	12 543 (3.4)	18 634 (5.0)
United States (MarketScan)	1 730 047	36 648 (2.1)	188 728 (10.9)	241 681 (14.0)
*Total*	3 856 041	56 809 (1.5)	470 749 (12.2)	570 855 (14.8)
**Ondansetron**				
Alberta	420 296	422 (0.1)	3293 (0.8)	5655 (1.3)
British Columbia	763 442	170 (0.0)	1998 (0.3)	2812 (0.4)
Manitoba	256 378	37 (0.0)	206 (0.1)	298 (0.1)
Ontario	102 701	33 (0.0)	67 (0.1)	122 (0.1)
Saskatchewan	213 433	32 (0.0)	306 (0.1)	482 (0.2)
*Canada overall*	1 756 250	694 (0.0)	5870 (0.3)	9369 (0.5)
United Kingdom (CPRD)	369 744	44 (0.0)	304 (0.1)	508 (0.1)
United States (MarketScan)	1 730 047	17 566 (1.0)	136 863 (7.9)	174 517 (10.1)
*Total*	3 856 041	18 304 (0.5)	143 037 (3.7)	184 394 (4.8)
**Doxylamine/pyridoxine**				
Alberta	420 296	1177 (0.3)	59 311 (14.1)	72 048 (17.1)
British Columbia	763 442	2089 (0.3)	107 102 (14.0)	116 166 (15.2)
Manitoba	256 378	842 (0.3)	36 652 (14.3)	42 673 (16.6)
Ontario	102 701	905 (0.9)	24 277 (23.6)	28 127 (27.4)
Saskatchewan	213 433	656 (0.3)	31 856 (14.9)	36 558 (17.1)
*Canada overall*	1 756 250	5669 (0.3)	259 198 (14.8)	295 572 (16.8)
United Kingdom (CPRD)	369 744	0 (0.0)	0 (0.0)	0 (0.0)
United States (MarketScan)	1 730 047	231 (0.0)	7497 (0.4)	9690 (0.6)
*Total*	3 856 041	5900 (0.2)	266 695 (6.9)	305 262 (7.9)

Pregnancy onset (the first day of the last menstrual period) in British Columbia, Manitoba, Ontario, Saskatchewan, and CPRD—between 2002 and 2014. Pregnancy onset in Alberta—between 2010 and 2014. Pregnancy onset in MarketScan—between 2007 and 2014.

^a^CPRD, United Kingdom Clinical Practice Research Datalink.

Antiemetic use varied by country (Table 2). Canada had the highest use during pregnancy (prescriptions were filled for 17.7% of all pregnancies), followed by the United States (14.0%), and the United Kingdom (medications were prescribed in 5.0% of all pregnancies). In Canada, doxylamine/pyridoxine was the most commonly used antiemetic during pregnancy (16.8% of pregnancies); other antiemetics, including ondansetron, were each used in less than 1% of pregnancies. In the United Kingdom, prochlorperazine was the most commonly used antiemetic (3.2% of pregnancies, S3 Table). In the United States, ondansetron was the most commonly used antiemetic (10.1% of pregnancies), followed by promethazine (4.7%, S3 Table).

**Fig 1:**
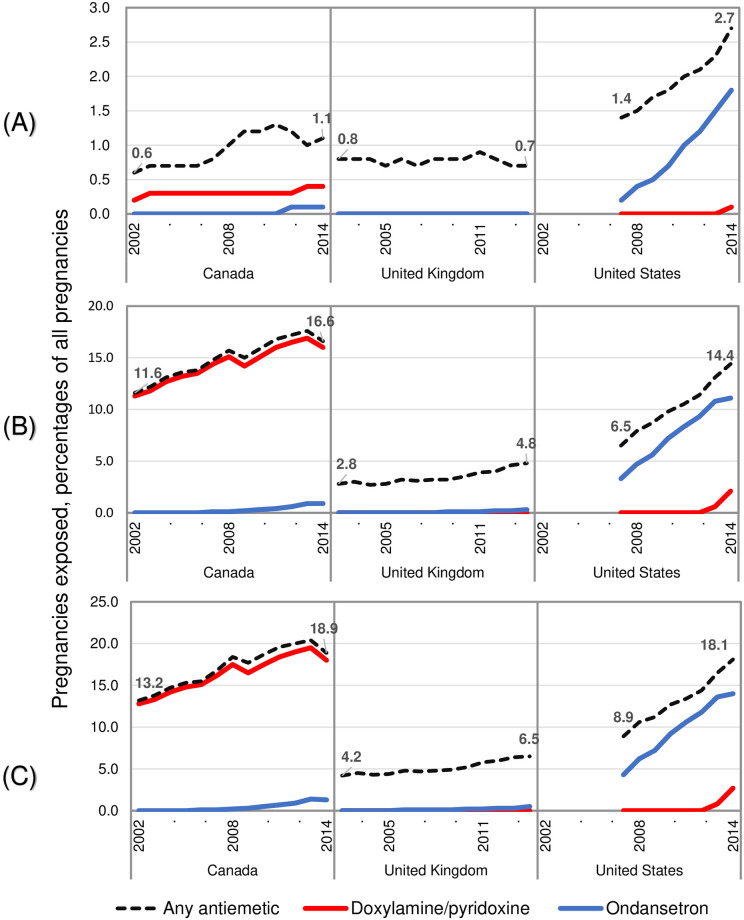
Use of antiemetic medications in Canada, the United Kingdom, and the United States between 2002 and 2014, by year of pregnancy onset. (A) in the 90 days before pregnancy onset; (B) during the first trimester of pregnancy; (C) anytime during pregnancy. Pregnancy onset was defined as the first day of the last menstrual period.

Trends in antiemetic use during pregnancy differed across jurisdictions. The use of any antiemetic medication anytime during the pregnancy increased from 2002 to 2014 in all countries (Fig 1). In Canada, the use of any antiemetic medication increased from 13.2% of pregnancies in 2002 to 18.9% in 2014; doxylamine/pyridoxine use increased from 12.8% to 17.9%, and ondansetron use increased from 0.0% to 1.2%. Similar trends were observed in the United States: the use of any antiemetic medication more than doubled from 8.9% of pregnancies in 2007 to 18.1% in 2014, ondansetron use increased from 4.3% to 14.0%, and doxylamine/pyridoxine use increased from 0 to 2.7% in 2014. In the United Kingdom, a more gradual increase in use of any antiemetic medication occurred, from 4.2% of pregnancies in 2002 to 6.5% in 2014. Ondansetron was rarely used in the United Kingdom (0.3% of pregnancies in 2014) and doxylamine/pyridoxine was not licensed there during the study period.

**Fig 2:**
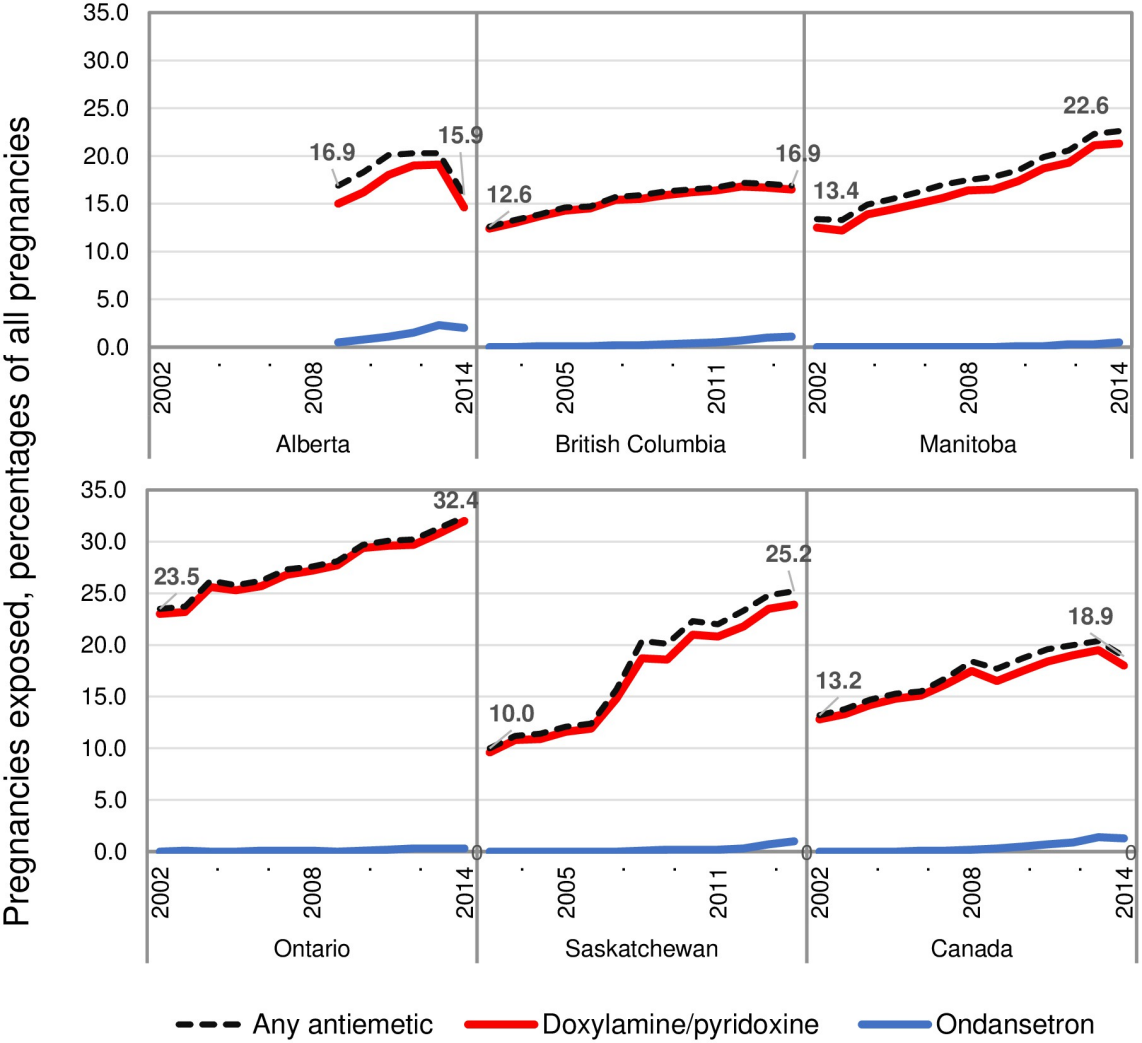
Use of antiemetic medications during pregnancy in Canada by province and year of pregnancy onset, 2002–2014. Pregnancy onset was defined as the first day of the last menstrual period.

Trends in antiemetic use over time varied among the Canadian provinces examined (Fig 2). Compared with the rest of Canada, a steep increase in use of any antiemetic medication occurred in Saskatchewan, especially between 2006 and 2008 (from 12.3% to 20.4% of pregnancies). In British Columbia, the increase over time was more gradual compared with the other provinces examined: 4.3% from 2002 to 2014, compared with an increase of between 9% and 15% during that time in Manitoba, Ontario, and Saskatchewan.

## Discussion

### Principal findings

This is the first study to compare patterns of antiemetic use during pregnancy in Canada, the United Kingdom, and the United States. The prevalence of antiemetic use during pregnancy, the type of antiemetic used, and the rate of increase in use over time all varied widely by jurisdiction. Some of this variation may be explained by differences in clinical guidelines [10,24,39,40], pre-existing patterns of practice (including the use of off-label medications) [28,31], regulatory approval, and/or drug formulary coverage [20,41]. For example, while the sustained-release combination product doxylamine/pyridoxine has been recommended as a first-line treatment for NVP in North America at least 15 years [11,20,22–24], the United Kingdom practice guidelines have recommended antihistamines and phenothiazines [40].

In the United States, we observed a slowing of rise in ondansetron utilization between 2012 and 2014. Factors influencing this trend could include a 2011 Food and Drug Administration Drug Safety Communication about a possible association between ondansetron treatment and QT interval prolongation [42] and the 2013 approval of the sustained-release combination product doxylamine/pyridoxine [11].

### Implications

The increase in antiemetic use during pregnancy observed in Canada was similar to that reported in two studies from 2002 to 2013 [43,44]. Also, the prevalence of doxylamine/pyridoxine use reported previously was similar to our data: 19.3% of births in Manitoba (between 2002 and 2013) [45], and 18% of births in British Columbia (in 2002) [43]. Previous studies conducted in three Canadian provinces (British Columbia, Manitoba, and Quebec between 2002 and 2013) demonstrated that doxylamine/pyridoxine was the most commonly used antiemetic during pregnancy [30,43,45–47].

In our study, prochlorperazine was the most commonly prescribed antiemetic in the cohort from the United Kingdom, and overall antiemetic use was low in that jurisdiction compared with use in North America. We used electronic medical records from primary care practices (CPRD) and did not capture prescriptions by midwives, obstetricians, or urgent care physicians. In addition, the slow-release combination product doxylamine/pyridoxine was not available in the United Kingdom during the study period, and it is possible that women used the over-the-counter immediate release products, which were not captured in our data. Fiaschi and colleagues recently reported a similarly low prevalence of NVP (9.0% of pregnancies) in the CPRD [48]. Two previous studies reported that prochlorperazine was the most commonly prescribed medication during pregnancy in the United Kingdom between 1988 and 2014 [48,49]. These findings demonstrate adherence with the Royal College of Obstetricians and Gynaecologists Green-top Guidelines that recommended the use of antihistamines and phenothiazines, including prochlorperazine, as the first-line treatment for NVP [40].

Similar to our study, these studies and others from the United States found that antiemetic use during pregnancy was increasing prior to 2015, driven largely by the use of ondansetron [26–28,31,50]. The prevalence of ondansetron use in the United States between 2000 and 2014 was 15.2% of pregnancies, and the highest use occurred during the first trimester [31,51]. In another study of pregnant women in the United States, both ondansetron and doxylamine/pyridoxine were the most commonly used antiemetic medications [52]. However, this study analyzed data from calls to a helpline, which may not be representative of the general population.

The increased use of antiemetics medications during pregnancy, especially ondansetron, is concerning given the possible harms associated with its use. Several studies have shown an increase in the risk of fetal death or congenital malformations in pregnancy exposed to ondansetron [51,53–55], but the results were inconsistent with other studies, including a recent study from our group [32]. Additionally, the variation between jurisdictions in antiemetic use over time may have important implications for women, newborns, and the healthcare system. It may be important to better understand the reasons for differences in the use of these medications between different jurisdictions to better plan health services for pregnant populations in different jurisdictions. These insights may also help improve diagnostic and prescribing practices.

### Strengths and limitations

This is the largest cohort study to date to describe patterns of antiemetic use during pregnancy in a geographically diverse population. We used administrative and clinical data that were systematically collected to reduce selection bias. This study is highly generalizable, as we used data from multiple jurisdictions.

The main limitation of our study was the use of administrative data, which may result in exposure misclassification. It is uncertain if all women who were dispensed antiemetics (or prescribed them in the CPRD data) actually took their medications. Second, because our dispensing records excluded drugs administered to inpatients, our results may underestimate the duration of exposure to antiemetics among women with more severe cases of NVP, who may be more likely to receive ondansetron. In the United Kingdom, we were unable to include prescriptions from obstetricians, gynecologists or midwives due to limitations of the CPRD data. Finally, we did not have access to information regarding over-the-counter purchases of doxylamine or pyridoxine. We also were missing data for gestational age in the Canadian and US databases, which required that we impute values for approximately 50% of the Canadian pregnancies and 100% of the pregnancies in the United States. This may have led to inaccuracies in determining cohort entry dates and exposure periods, despite using methods that were previously validated [56,57].

## Conclusions

In this large international study of antiemetic use during pregnancy, we observed increasing use of antiemetics over time and varying prevalence of use between countries and within Canada. This included variation in the prevalence of antiemetic use to treat NVP, the choice of antiemetic used, and the rate of increase in antiemetic use over time. The variation and increasing antiemetic use over time may have important implications for women, newborns, and the healthcare system. Continued monitoring of antiemetic use and further research are important to better understand the reasons for differences in the use of these medications between different jurisdictions. Furthermore, research on the safety of these medications is important to be better informed on the risk–benefit balance of antiemetic treatment during pregnancy.

## Supporting information

S1 Table Diagnostic, procedure and fee codes used to identify pregnancy outcome. (PDF)

S2 Table Pregnancy cohort: Record flow. (PDF)

S3 Table Three most commonly used antiemetic medication in pregnant women, by jurisdiction. (PDF)

S4 Table Minimal data set: The use of antiemetic by jurisdiction, year, and antiemetic medication. (PDF)

S1 File PLOS’ questionnaire on inclusivity in global research. (PDF)

## Abbreviations

CNODES: Canadian Network for Observational Drug Effect Studies
CPRD: Clinical Practice Research Datalink
NVP: Nausea and vomiting of pregnancy
