# Supplementary material for: Patterns of antiemetic medication use during pregnancy: A multi-country retrospective cohort study
Source: PLoS One. 2022 Dec 1;17(12):e0277623. doi: 10.1371/journal.pone.0277623 (PMC9714905; doi:10.1371/journal.pone.0277623)
Supplement: S1 Table — (PDF) [file pone.0277623.s001.pdf]

S1 Table. Diagnostic, procedure and fee codes used to identify pregnancy outcome

Outcome	ICD-9 codes[a]	ICD-10 codes[a]	Other
Live birth	V27.0, V27.2, V27.5	Z37.0, Z37.2, Z37.5	
Stillbirth<br><br>(including mixed/unspecified birth)	V27.1, V27.4, V27.7, 656.4<br><br>V27.3, V27.6, V27.9	O36.4, Z37.1, Z37.4, Z37.7<br><br>Z37.3, Z37.6, Z37.9	Database-specific ambulatory fee-for-service codes
Induced abortion	635, 636	O04	Database-specific ambulatory fee-for-service codes
Spontaneous abortion	634, 630, 631, 632, 633, 637	O03, O01, O02, O05	Database-specific ambulatory fee-for-service codes

[a] ICD: International Classification of Diseases. In the United Kingdom Clinical Practice Research Datalink, we used the Pregnancy Register outcome variable to identify pregnancy outcomes. These outcome variables were derived using an algorithm based on Read codes recorded in the CPRD.
