# Supplementary material for: Patterns of antiemetic medication use during pregnancy: A multi-country retrospective cohort study
Source: PLoS One. 2022 Dec 1;17(12):e0277623. doi: 10.1371/journal.pone.0277623 (PMC9714905; doi:10.1371/journal.pone.0277623)
Supplement: S2 Table — (PDF) [file pone.0277623.s002.pdf]

S2 Table. Pregnancy cohort: record flow

Jurisdiction	Canada					United Kingdom (CPRD[a])	United States (MarketScan)	Total
	Alberta	British Columbia	Manitoba	Ontario	Saskatchewan			
Source population: number of women	1,342,677	2,082,055	573,711	4,861,333	521,843	467,598	37,154,585	47,003,802
Number of pregnancy records[b]	820,080	2,850,734	697,826	2,956,997	606,352	731,131	5,607,950	14,271,070
After excluding pregnancies with more than two different outcomes	746,047	2,568,690	697,826	2,885,958	516,168	724,969	5,530,707	13,670,365
After excluding pregnancies with insufficient length of enrollment	746,047	2,425,294	657,214	2,722,332	475,066	653,851	3,023,208	10,703,012
After excluding pregnancies based on age	742,203	2,423,732	657,176	2,722,106	475,043	653,831	3,022,140	10,696,231
After excluding overlapping pregnancies	448,567	823,184	276,654	2,371,141	231,287	635,340	1,839,326	6,625,499
After applying other site-specific exclusion criteria[c]	448,567	823,184	276,654	109,179	231,287	387,459	1,834,010	4,110,340
Final cohort[d]	420,296 (10.9)	763,442 (19.8)	256,378 (6.6)	102,701 (2.7)	213,433 (5.5)	369,744 (9.6)	1,730,047 (44.9)	3,856,041 (100.0)

[a]CPRD, United Kingdom Clinical Practice Research Datalink.

[b]All data sources (April 2002–March 2016).

[c]The site-specific exclusion criteria were as follows: (1) in Ontario, a woman had to have had at least one drug claim in the 6 months AND 6–12 months before the pregnancy outcome date to enter the cohort; this is a proxy for drug coverage; (2) in the CPRD database, women without a link to hospital data (Hospital Episode Statistics) were excluded; and (3) in the MarketScan data, women were excluded if their enrollment ended or they had enrollment gaps before the pregnancy outcome date.

[d] Final cohort after excluding pregnancies with cohort entry before 2002 and after 2014. Number of pregnancies (% of the total).
