# Supplementary material for: Patterns of antiemetic medication use during pregnancy: A multi-country retrospective cohort study
Source: PLoS One. 2022 Dec 1;17(12):e0277623. doi: 10.1371/journal.pone.0277623 (PMC9714905; doi:10.1371/journal.pone.0277623)
Supplement: S3 Table — (PDF) [file pone.0277623.s003.pdf]

S3 Table. Three most commonly used antiemetic medication in pregnant women, by jurisdiction

Jurisdiction	Antiemetic medication	Percentages from pregnancies exposed
Alberta	Doxylamine + pyridoxine	17.1
	Dimenhydrinate	1.6
	Ondansetron	1.3
British Columbia	Doxylamine + pyridoxine	15.2
	Metoclopramide	0.7
	Ondansetron	0.4
Manitoba	Doxylamine + pyridoxine	16.6
	Dimenhydrinate	1.4
	Metoclopramide	0.9
Ontario	Doxylamine + pyridoxine	27.4
	Metoclopramide	0.8
	Prochlorperazine	0.4
Saskatchewan	Doxylamine + pyridoxine	17.1
	Dimenhydrinate	1.1
	Metoclopramide	1.0
United Kingdom	Prochlorperazine	3.2
	Promethazine	1.7
	Metoclopramide	1.7
United States	Ondansetron	10.1
	Promethazine	4.7
	Metoclopramide	1.5
