# Supplementary material for: Patterns of antiemetic medication use during pregnancy: A multi-country retrospective cohort study
Source: PLoS One. 2022 Dec 1;17(12):e0277623. doi: 10.1371/journal.pone.0277623 (PMC9714905; doi:10.1371/journal.pone.0277623)
Supplement: S4 Table — (PDF) [file pone.0277623.s004.pdf]

S4 Table. Minimal data set: The use of antiemetic by jurisdiction, year, and antiemetic medication

Jurisdiction	Entry Year	Total number of pregnancies	Percentages of pregnancies exposed to antiemetic between the cohort entry date and the pregnancy outcome date		
			Ondansetron	Doxylamine + pyridoxine	Any antiemetic medication
Alberta	2008	26348	0.5	19.0	21.0
	2009	69315	0.5	15.0	16.9
	2010	70508	0.8	16.2	18.3
	2011	71249	1.1	18.0	20.1
	2012	74247	1.5	19.0	20.3
	2013	79529	2.3	19.1	20.3
	2014	55448	2.0	14.6	15.9
British Columbia	2002	55375	0	12.4	12.6
	2003	56013	0	13.0	13.3
	2004	56679	0.1	13.7	13.9
	2005	56814	0.1	14.3	14.6
	2006	58848	0.1	14.5	14.7
	2007	60254	0.2	15.4	15.7
	2008	61017	0.2	15.5	15.9
	2009	60030	0.3	15.9	16.3
	2010	60511	0.4	16.2	16.5
	2011	59031	0.5	16.4	16.7
	2012	60126	0.7	16.8	17.2
	2013	59290	1.0	16.7	17.1
	2014	59454	1.1	16.5	16.9

Jurisdiction	Entry Year	Total number of pregnancies	Percentages of pregnancies exposed to antiemetic between the cohort entry date and the pregnancy outcome date		
			Ondansetron	Doxylamine + pyridoxine	Any antiemetic medication
Manitoba	2002	18133	0	12.5	13.4
	2003	18337	0	12.2	13.3
	2004	17773	0	13.9	14.9
	2005	17983	0	14.4	15.5
	2006	19768	0	15.0	16.2
	2007	19830	0	15.6	17.0
	2008	20345	0	16.4	17.5
	2009	20362	0	16.5	17.8
	2010	20481	0.1	17.4	18.5
	2011	20499	0.1	18.7	19.9
	2012	20866	0.3	19.3	20.6
	2013	20906	0.3	21.1	22.3
	2014	21095	0.5	21.3	22.6
Ontario	2002	7307	0	23.0	23.5
	2003	7606	0.1	23.2	23.7
	2004	7756	0	25.6	26.2
	2005	7897	0	25.3	25.8
	2006	8198	0.1	25.7	26.2
	2007	8214	0.1	26.8	27.3
	2008	8128	0.1	27.2	27.6
	2009	8474	0	27.7	28.1
	2010	8625	0.1	29.4	29.7
	2011	8280	0.2	29.6	30.1
	2012	7800	0.3	29.7	30.2

Jurisdiction	Entry Year	Total number of pregnancies	Percentages of pregnancies exposed to antiemetic between the cohort entry date and the pregnancy outcome date		
			Ondansetron	Doxylamine + pyridoxine	Any antiemetic medication
	2013	7441	0.3	30.8	31.3
	2014	6975	0.3	32.0	32.4
Saskatchewan	2002	14562	0	9.6	10.0
	2003	14787	0	10.8	11.2
	2004	14943	0	10.9	11.4
	2005	15301	0	11.6	12.1
	2006	15606	0	11.9	12.4
	2007	16260	0	14.8	15.7
	2008	16560	0.1	18.7	20.4
	2009	17082	0.2	18.6	20.1
	2010	17094	0.2	21.0	22.3
	2011	17149	0.2	20.8	22.0
	2012	17865	0.3	21.8	23.3
	2013	18044	0.7	23.5	24.8
	2014	18180	1.0	23.9	25.2
Canada	2002	95377	0	12.8	13.2
	2003	96743	0	13.3	13.8
	2004	97151	0	14.2	14.7
	2005	97995	0	14.8	15.3
	2006	102420	0.1	15.1	15.5
	2007	104558	0.1	16.2	16.8
	2008	132398	0.2	17.5	18.4
	2009	175263	0.3	16.5	17.7
	2010	177219	0.5	17.5	18.7

Jurisdiction	Entry Year	Total number of pregnancies	Percentages of pregnancies exposed to antiemetic between the cohort entry date and the pregnancy outcome date		
			Ondansetron	Doxylamine + pyridoxine	Any antiemetic medication
	2011	176208	0.7	18.4	19.6
	2012	180904	0.9	19.0	20.0
	2013	185210	1.4	19.5	20.4
	2014	161152	1.3	18.0	18.9
United Kingdom	2002	23856	0	0	4.2
	2003	27194	0	0	4.5
	2004	28565	0	0	4.3
	2005	30092	0	0	4.4
	2006	30989	0.1	0	4.8
	2007	31871	0.1	0	4.7
	2008	32586	0.1	0	4.8
	2009	32549	0.1	0	4.9
	2010	31447	0.2	0	5.2
	2011	30673	0.2	0	5.8
	2012	27792	0.3	0	6.0
	2013	23617	0.3	0	6.4
	2014	18513	0.5	0	6.5
United States	2006	33942	3.3	0	7.8
	2007	180663	4.3	0	8.9
	2008	205299	6.2	0	10.6
	2009	236204	7.2	0	11.2
	2010	224447	9.2	0	12.7
	2011	236236	10.6	0	13.4
	2012	228625	11.8	0	14.4

Jurisdiction	Entry Year	Total number of pregnancies	Percentages of pregnancies exposed to antiemetic between the cohort entry date and the pregnancy outcome date		
			Ondansetron	Doxylamine + pyridoxine	Any antiemetic medication
	2013	218947	13.6	0.8	16.5
	2014	199626	14.0	2.7	18.1
